# Health is a political choice: why conduct healthcare research? Value, importance and outcomes to policy makers

**DOI:** 10.1186/s40504-020-00100-8

**Published:** 2020-07-27

**Authors:** M. Walid Qoronfleh

**Affiliations:** Research & Policy Department, World Innovation Summit for Health (WISH), Qatar Foundation, P.O. Box 5825, Doha, Qatar

**Keywords:** Eastern Mediterranean region, EMR, Healthcare research, Evidence-based research, Politics, Health systems, Public health policy, National policy, Health and social outcomes, Qatar

## Abstract

This paper offers the Eastern Mediterranean Region (EMR) viewpoint with Qatar as a case for lasting transformation of health systems. The Qatar case study illustrates the importance of research in the development of health policy. It provides description of a series of projects that have been undertaken in relevant national areas such as autism, dementia, genomics, palliative care and patient safety. The paper discourse draws attention to investment requirement in health research systems to respond to country national health priorities and to strengthen public health policies for improving health and social outcomes by narrowing the gap between research and politics. In short, the discussion highlights the following: i) health is a human right marching towards universal health care, with research underpinning every advance in health care and quality medical services; ii) evidence-based research is emerging as a critical tool to aid policy- and decision-makers; iii) investment necessity in healthcare research/systems to enable responding to a country’s national health priorities and to strengthen public health policies; and iv) need for multi-sectoral involvement of stakeholders to bridge the gap between research and politics. Finally, atypical stakeholders’ engagement and bond to politics is a prerequisite to achieve healthcare objectives and policy success so as to reap the benefits of public health results.

## Introduction

Historically between the 8th - 14th century, The Eastern Mediterranean Region (EMR, as defined by the WHO) had a remarkable influence on health care research and systems for a string of many firsts in diagnosis, treatment and patient management including many scientific discoveries (Majeed 2005; Pallejà de Bustinza 2016). Today, in the EMR non-communicable diseases account for 75% of disease burden having the highest traits of risk factors as well such as obesity, smoking and sedentary lifestyle, thusly, increasing mortality amongst the population. Indeed, the World Health Organization (WHO) statistics speaks vividly of this challenge (Fouad et al. 2018). Definitely, there are major health and development challenges facing the EMR. Amongst these chief challenges are resource constraints, regional conflicts, and inadequate health systems research that compound and exacerbate many issues confronting EMR, all of which make for uncertain future for the region. It is about time to re-envisage health systems. It is critical to breakdown silos and boundaries, re-orientate health systems to address public health and the broader ecosystem influencing health.

This treatise presents an EMR perspective with Qatar as an example for long-term transformation of health systems. The discourse highlights investment in health research systems (HRS), how HRS respond to country healthcare priorities and discussing the broader influence on EMR. Equally important, how essential and imperative is research/gained knowledge in the development of health policy.

## Healthcare research systems

Health research is very important and has high value to both individuals as well as society. Overall, healthcare research can contribute to information about disease trends and risk factors, outcomes of treatment or public health interventions, functional abilities, patterns of care, and health care costs and use. Different healthcare research types provide complementary insights. For instance, clinical trials can provide critical evidence about the efficacy and adverse effects of therapeutic treatment; also, crucial post-market surveillance data for comparing and improving the use of drugs, vaccines, medical devices, and diagnostics. Therefore, clinical experience at a population level is imperative for identifying relatively rare adverse effects and for determining the effectiveness in different sub-population groups (stratification) leading to precision medicine and individualized therapy. Ultimately, this allows the actual development of clinical guidelines for best practices and to ensure high-quality patient care.

### Clinical research

Clinical healthcare research have led to significant discoveries, the development of novel therapies, and a remarkable improvement in health care and public health. Economists have found that medical research can have an enormous impact on human health, wellbeing and longevity, and that the resulting increased productivity of the population contributes greatly to the national economy (Hatfield et al. 2001; Murphy and Topel 1999) besides the obvious individual benefits of improved health. If the research enterprise is impeded, or if it is less robust, vital societal interests are affected. Clinical research and clinical trials registry are yet to be fully established in most of the EMR countries or have formulated laws governing the conduct of clinical trials. Moreover, system maturity vary between EMR countries. The Middle East North Africa (MENA) region is predicted to be one of the leading regions for clinical research outsourcing due to the availability of essential infrastructure for the conduct of clinical trials, access to required patients and financial benefits.

### Public health research

Public healthcare research with a particular focus on health services research is another approach to provide information and practical knowledge to improve health outcomes and return on investment. For example, the development of Herceptin, a breast cancer treatment, is a prime illustration of the benefits of healthcare research using biological specimens and patients’ medical records (Slamon et al. 1987). Other examples of findings from healthcare research have changed the practice of medicine as well. A case in point, research underlying estimated patient’s death from medical errors in hospitals, which has provided valuable proof for reducing these medical errors by implementing health information technology like e-prescribing (Bates et al. 1998). Furthermore, medical records research has demonstrated that preventive screening services (e.g., mammography) substantially reduce mortality and morbidity at very reasonable cost (Mandelblatt et al. 2003). Similar kinds of investigation has also established a correlation between nursing shortage and patient health outcomes by documenting that patients in hospitals with fewer registered nurses stay in hospital longer, thus, are more likely to suffer complications the likes of urinary tract infections, upper gastrointestinal bleeding and nosocomial infections (Needleman et al. 2002) resulting in increased hospitalization cost. These findings have all informed and influenced policy decisions at the national level with effective change to medical practice, cost containment and improved outcomes. As the use of electronic medical records increases, the pace of this form of research is accelerating, and the opportunities to generate new knowledge about what works in health care are expanding (Coalition for Health Services Research (CHSR) 2008). Poor health information system has been identified as a major challenge in the EMR healthcare system. Even though, EMR countries have embraced healthcare technology advances in the last decade along with tremendous investment in healthcare IT products and services in order to drive higher adoption of electronic and digital healthcare systems. However, computer and English literacy remain an issue. The available evidence indicate that the region still lags behind and that there were many factors that hindered the widespread adoption such as cost, procurement and maintenance.

## Healthcare research and policy

Health is a human right, with research underpinning every advance in health care. The goal is to attain universal healthcare coverage. Undeniably, better research, i.e., evidence-based yields better health. Evidence-based research is emerging as a critical tool to policy makers. Increasingly, quality research that is backed up by caliber data/information and superior analytics is being utilized by policy and decision makers to formulate, enact and improve government policies thus influencing a country’s health status and population health. A case in point, Europe leads in many areas of research and has developed powerful models of cross-border, cross-sectoral research cooperation. The Scientific Panel for Health (SPH) is a science-led stakeholder platform, which elaborates scientific input concerning this societal challenge. It assists the EU Commission in the preparation of legislative proposals and policy initiatives. This expert led group has proposed the creation of European Council for Health Research to provide a comprehensive policy for health research in Europe and facilitate cross-border collaboration (The Lancet 2016). It is anticipated that these actions would lead to improved overall health outcomes and health economics, in other words better outcomes related to efficiency, effectiveness, value and behavior in the production and consumption of health and healthcare.

### The Eastern Mediterranean region (EMR)

Since the inception of the first Global Symposium on Health Systems Research and the era of Sustainable Development Goals (SDG) the goal of ‘*leaving no one behind*’ is falling behind. The intertwined complexities and issues facing EMR require an evolution from primary focus on health to a broader focus on SDGs’ and multi-sectoral involvement. Therefore, strengthening science, research, policies and societal engagement for the EMR countries is critical to ensure dialogue continuity and experience healthcare improvements at all levels. Continued investment in HRS not only is essential but also is an enabler to equip countries to deliver on promises and resolve increasingly complex and interconnected health and development challenges of the twenty-first century. Devoting time and resources to integrated knowledge translation and engaging atypical stakeholders is a fundamental paradigm shift in the way we think about health systems. This demands being self-critical of the systems, to engaging with the social determinants of health to appealing to health systems influencers such as political and financial intuitions. EMR mandatory achievement pillars are strengthening public health policy and improving health outcomes by narrowing the gap between research and politics.

### Qatar

Finally, WISH, its research team and the Qatari health research community at large are working to communicate and educate the public about why healthcare research is important and how healthcare research is done. Equally important, healthcare researchers must convey the value of health care improvements derived from public health research, quality of medical records, availability of biological samples and to stress the negative impact of incomplete datasets/analysis on research findings vis-à-vis public health. In this vein, the Qatar Ministry of Public Health (MoPH) organized its First Biennial Qatar Public Health Conference (QPHC) from 18 to 19 November 2019 at the Ritz Carlton Hotel | Doha, Qatar. The MoPH Public Health Department has organized QPHC to convene every 2 years to discuss distinct public health themes with insights from national, regional and international experts.

In preparation for the FIFA World Cup 2022, which will be held in Qatar, Qatar Health 2020 – a first of its kind healthcare conference aimed at improving healthcare professionals’ understanding of mass gathering events and informing policies. The conference - a collaborative effort between Hamad Medical Corporation (HMC) and MoPH - took place from 16 to 18 January 2020 at the Sheraton Grand Doha Resort and Convention Hotel | Doha, Qatar. The COVID-19 pandemic incidence is a stark reminder of the risk threat posed by communicable disease outbreaks including spread at major sporting events.

## Evidence-based research contribution to policy – case study examples from Qatar

The World Innovation Summit for Health (WISH) and the research team are committed to positively influencing public health research and policies in Qatar. These areas include autism, mental health, dementia, patient safety, non-communicable diseases (cancer, diabetes & obesity), precision medicine and bioethics. WISH endeavors to address Qatar’s national healthcare priorities. The environmental context is described below. Additionally, it elaborates on the importance of multi-sectoral engagement to galvanize the community to work around silos and boundaries demonstrating appreciation to the influence and proper understanding of the Qatari culture and business norms.

### Qatari context

Similar to other countries in the region, Qatar has witnessed a rapid change in many aspects of life over the past four decades. Primarily because of rapid urbanization and socioeconomic development following the region’s “oil boom” that took place between 1970 and 1980, Qatar’s population today enjoys a very different standard and way of life compared to earlier generations. Qatar has recently emerged as one of the wealthiest countries in the world when measured in terms of gross domestic product (GDP) per capita (The Heritage Foundation 2019). Among other indicators, great progress is being observed when it comes to the country’s health data, which reveals an increasing life expectancy and a significant drop in infant mortality. Qatar has achieved overall better health outcomes over the past several decades due to making significant investments in the healthcare infrastructure. The state boasts the highest life expectancy rate in the World Health Organization’s Eastern Mediterranean Region (EMRO), and globally ranks in the top 25th percentile for healthcare access and quality. Qatar’s healthcare expenditure and investment is also among the highest in the Middle East, with QR 22.7 billion (USD $6.2 billion) invested in 2018. These achievements have led some organizations to rank Qatar fifth (5th) in the world for healthcare (Legatum Institute 2018). Certainly, Qatar has the financial prowess with a budding health care system. The healthcare system, healthcare infrastructure and public health policies continue to be on a developing trajectory towards an ambitious personalized healthcare goal.

In 2018, the MoPH-Qatar launched its second National Health Strategy (NHS, 2018–2022 *aka* NHS 2.0), which signified an important step in the State’s health system development. The second National Health Strategy was developed taking into account the United Nations (UN) Agenda for Development, the WHO’s recommendations and objectives, and the regional context. The NHS 2.0 has established three main objectives: better health, better care, and better value. Priority areas include the development of an integrated model of high-quality care and service delivery for the State of Qatar; enhanced health promotion and disease prevention; enhanced health protection; health integrated across the country in all policies; and establishing effective systems of health governance and leadership.

The legendary management consultant and writer Peter Drucker is famously quoted “*Culture eats strategy for breakfast*”. These developments and achievements would not have been possible without convening key opinion and thought leaders, developing a global health viewpoint, and committing to encourage on evidence-based research. A multisectoral approach that is culturally sensitive is powerful and empowering. It is a surer route to success.

## Case studies - contributing to Qatar national policy

The Qatar case study series illustrates the significance of research in the development of health policy. Research is at the core of everything WISH does. WISH is continuing its contribution to improve Qatar’s healthcare system. Research undertaken by WISH has made a significant influence to national policies in Qatar. Few examples are highlighted below that address Qatar’s national priorities and are in alignment with MoPH national health strategies. The specific projects case studies show the importance of application of knowledge coming from research. Further, they elucidate and emphasize the link between health, society and policy to improve both health and social outcomes. The diverse stakeholders’ participation ensures alignment, endorsement and drives action. This is exemplified in the underneath narrative.

### Dementia and autism

The social and economic burden of dementia is clear and enormous even today. The staggering increase in the prevalence of autism spectrum disorder (ASD) over the past 40 years is well document. Both have severe effects on the quality of life of individuals and causes tremendous strain on caregivers and healthcare systems. The WISH dementia reports [*A CALL TO ACTION: The Global Response to Dementia through Policy Innovation, 2015*; *Enhancing the Response to the Burden and Impact of Dementia, 2016*] (Qoronfleh 2017), and the WISH autism report [*AUTISM: A Global Framework for Action, 2016*] have provided the foundation of evidence based knowledge to frame the National Dementia Strategy and the National Autism Strategy, which were launched in 2018 and 2017, respectively. In addition, the 2015 policy report on dementia has provided a vehicle for the country’s involvement in the WHO Global Dementia Observatory that continues to this day. WISH has also implemented the report findings through pioneering community outreach and awareness programs such as providing more social and recreational services to Qatar’s autism community (Qoronfleh et al. 2019).

### Patient safety

Estimates show that in high-income countries as many as 1 in 10 patients is harmed while receiving hospital care. Patient death occurring due to a preventable medical accident, while receiving health care, is estimated to be 1 in 300. Approximately two- thirds of all adverse events occur in low and middle-income countries (sources WHO August 2019). One of the key policy recommendations from the WISH 2015 report on patient safety [*Transforming Patient Safety: a Sector-Wide Approach, 2015*] was focused around the need and urgency of including patient safety in the curriculum of medical/clinical majors. Following that, WISH collaborated with MedStar Health, USA and MoPH to bring the Academy for Emerging Leaders in Patient Safety to Qatar on an annual basis and to conduct a capacity building program, an inter-professional intensive training for both faculty members and students on reducing medical errors in the hospital setting. Since its inception in 2016, over 250 participants from all health science colleges around Qatar have graduated from the program. WISH patient safety framework and Qatari experience were also on display at two international conferences. At the 8th International Patient Safety Conference 13–14 September 2019, Novotel HICC, Hyderabad, India and at the 4th International Conference on Patient Safety 30 November – 1 December, 2019 organized by Riphah Institute of Healthcare Improvement & Safety and hosted by Rawalpindi Medical University (RMU), Rawalpindi, Pakistan. Hence, stressing the worth and benefit of this work beyond the local public to influencing the global healthcare community especially for recognized countries with resource constraints.

### Accountable care

Health systems around the world are facing rising health expenditures due to aging populations with complex conditions, new medical technologies, and challenges in providing universal access to care. Consequently, policymakers are developing more person-centered care models, and “value-based” payment reforms to support and sustain them. Many health systems are implementing accountable care – defined as a group of providers who are held jointly accountable for achieving a set of outcomes for a defined population over a period of time and for an agreed cost – to adopt the necessary organizational competencies and health policies needed to implement innovations in care that support better population health while avoiding unnecessary costs. In 2016, WISH led the efforts in establishing a multidisciplinary task force to implement an integrated care model between Primary Health Care Corporation (PHCC) and HMC, which was published in its forum report [*Implementing Accountable Care to Achieve better Health at a Lower Cost, 2016*]. The pilot phase of the project resulted in the SMART clinic project, a national diabetes screening and prevention program. The pilot demonstrated how a health system could begin implementing accountable care by reorganizing care delivery before addressing payment reform. This model has now been expanded and adapted by PHCC and HMC to tackle screening and prevention of other chronic diseases evolving into the “*The Better Together Program*” besides being incorporated into the NHS 2.0 strategy. Qatar’s National Health Strategy, 2018–2022, highlights the need for creating quality, care delivery infrastructure – so-called “high-value health systems” – to enable such improvements in patient access, affordability, and outcomes. The latest policy and outcome developments facilitated by WISH were discussed in a roundtable on the 14th of November 2018 at QNCC in Doha with Duke University Center for Health Policy and PHCC leadership.

### Genomics and ethics

Given the foreseeable scientific leap in genomics in the gulf region, the need for a solid knowledge base pairing scientific research with cutting-edge research in Islamic ethics is more urgent than ever. Managing the promising, pioneering genomics outcome coupled with the potential ethical challenges is a conundrum, one that requires acknowledging and understanding the religio-cultural fabric of this region and the wider Muslim world to which it belongs (Al-Dewik and Qoronfleh 2019). Religion and socio-cultural ethical conduct not only influence research but also shapes political/policy perspectives. In 2018, the Qatar National Research Ethics Committee published the country’s very first “Genomic Policy” that aims at assisting investigators in the design and conduct of genomic research, oversees genomic research activity and addresses the different associated risks. This was based on the policy recommendations that stemmed from the report that was published in 2016 by WISH entitled *Genomics in the Gulf Region and Islamic Ethics, 2016*.

### Palliative care

Palliative care (PC) is a relatively new medical specialization that embodies a number of universally shared values. Its principal aims are to relieve pain and other distressing symptoms, improve quality of life for individuals living with serious illness, and provide patients with good end-of-life care. PC physicians face various ethical dilemmas. Experts predict a rise in the demand for PC. There are various reasons for this, such as the increase of geriatric populations and prevalence of chronic and life-limiting diseases, which affect a population regardless of age. To provide culturally sensitive PC, patients’ (religious) beliefs and moral worlds must be integral parts of the care package. PC is an integral part of healthcare, and many countries in the Arab/Islamic world are increasingly offering this care. This is an area of particular sensitivity and interest within the region including the State of Qatar, and represents a growing need for both clinicians and family members to have access to culturally acceptable guidance, which would alleviate suffering and facilitate compassion. Through the WISH policy report [*Palliative Care and Islamic Ethics: exploring key issues and best practice, 2018*], the proposed guidelines are under consideration, and should stimulate and galvanize efforts at the national as well as the regional levels. WISH has recently launched an awareness campaign around palliative care practice both regionally and nationally.

WISH and the Pontifical Academy for Life (Vatican City) jointly organized a two-day symposium on religion and medical ethics 11–12 December 2019. This special symposium examined the role that religion plays in providing holistic care in the context of medical ethics. By focusing on the intersection of belief-based and evidence-based approaches to care, speakers and participants had the opportunity to highlight and explore the benefits of interdisciplinary and interfaith approaches to treating the body, mind and soul. The two main topic areas of healthcare were Palliative Care and The Mental Health of the Elderly. The aim was to harness intellectual input, deliberations, and to produce a special report with a framework and/or action plan with policy recommendation to facilitate national policy composition or guidelines to healthcare facilities that are integral to holistic care including workshops development and training sessions for healthcare workers. A vivid example of robust cross-cultural interaction and interfaith dialogue.

### Nursing now

Effective and high-quality health systems rely on multidisciplinary teams. Nurses and midwives play a central role in all health systems. The WHO has declared that 2020 is the “Year of the Nurse”. In 2018, WISH published a report on nursing and midwifery [*Nursing and Midwifery the key to the rapid and cost-effective expansion of high quality universal health coverage, 2018*]. This report is about the contribution of both nurses and midwives to universal health coverage (UHC). Additionally, in partnership with the MoPH, WISH has been instrumental in the establishment of Nursing Now Qatar. Indeed, Nursing now Qatar was one of the very first to be launched in the MENA region and has firmly put Qatar at the vanguard of this global campaign. While still in its relative infancy, this program will address significant workforce issues and capacity building including promoting the profession and encouraging retention, both now and in the future, around nursing and midwifery in Qatar. WISH also welcomed representatives from countries across Asia, Africa, and the Middle East and conducted a two-day symposium for nursing leaders on Health Workforce Data on 11 April 2019 at QNCC, Doha, Qatar. The workshop was in consultation with HMC and WHO. Afterwards, WISH has partnered with the global Nursing Now Campaign and the International Council of Nurses (ICN) offering a nursing symposium to train young nursing leaders from around the world to amplify their voices and positively influence healthcare policy, and underscore their role in UHC ahead of attending the annual World Health Assembly in Geneva, Switzerland.

## Conclusions

Health is a human right, with research underpinning every advance in health care. Undoubtedly, better research that is evidence-based yields better health. The EMR is facing considerable challenges. A game changer approach is the broad, multi-sectoral involvement of stakeholders to bridge the gap between research and politics. This leads to strengthening public health policy and improving health/social outcomes. Evidence-based research is emerging as a critical tool to policy makers. This type of research should be more accessible to policymakers. For instance, The Sixth Global Symposium on Health Systems Research (HSR2020) served as a catalytic platform for sharing knowledge and experiences, raising awareness and advocating for change, building capacity, and developing strategic partnerships to address the challenges facing health and development today.

WISH, its research team, and the Qatari health research community at large are working to educate the public about why healthcare research is important and how healthcare research is done. Equally important, healthcare researchers must convey the value of health care improvements derived from public health research, ensuring a high quality of medical record keepings, and through the availability of biological samples, and to stress the negative impact of incomplete datasets/analysis on research findings *vis-*à-*vis* public health, and use of evidence-based research in policy formulation as well as intervention. WISH is addressing Qatar’s national priorities that are in alignment with MoPH national health strategies.

In sum, lessons learned to implement policy recommendations or enact successfully policies include:

i) Recognize that health is a human right to achieve universal health care, with research underpinning every advance in health care,
ii) Execute evidence-based research, it is a critical tool to policy- and decision makers,
iii) Invest in health research systems to respond to country national health priorities, strengthen public health policies including their outcomes and deliver quality medical services, and
iv) Involve multi-sectoral stakeholders to bridge the gap between research and politics.

Finally, it is our firm belief that atypical stakeholders’ engagement and bond to politics is fundamental to achieve healthcare objectives and policy success so as to reap the benefits of public health results.

## Data Availability

All data generated or analyzed during this study are included in this published article.

## Abbreviations

EMR: Eastern Mediterranean Region
GDP: Gross Domestic Product
HMC: Hamad Medical Corporation
HRS: Health Research Systems
MENA: Middle East North Africa
MoPH: Ministry of Public Health-Qatar
PC: Palliative Care
PHCC: Primary Health Care Corporation
SDG: Sustainable Development Goals
UHC: Universal Health Coverage
UN: United Nations
WHO: World Health Organization
WISH: World Innovation Summit for Health
